# Magnetoresistance and robust resistivity plateau in MoAs_2_

**DOI:** 10.1038/s41598-017-15962-w

**Published:** 2017-11-15

**Authors:** Jialu Wang, Lin Li, Wei You, Tingting Wang, Chao Cao, Jianhui Dai, Yuke Li

**Affiliations:** Department of Physics and Hangzhou Key Laboratory of Quantum Matter, Hangzhou Normal University, Hangzhou, 310036 China

## Abstract

We have grown the MoAs_2_ single crystal which crystallizes in a monoclinic structure with C2/m space group. Transport measurements show that MoAs_2_ displays a metallic behavior at zero field and undergoes a metal-to-semiconductor crossover at low temperatures when the applied magnetic field is over 5 T. A robust resistivity plateau appears below 18 K and persists for the field up to 9 T. A large positive magnetoresistance (MR), reaching about 2600% at 2 K and 9 T, is observed when the field is perpendicular to the current. The MR becomes negative below 40 K when the field is rotated to be parallel to the current. The Hall resistivity shows the non-linear field-dependence below 70 K. The analysis using two-band model indicates a compensated electron-hole carrier density at low temperatures. A combination of the breakdown of Kohler’s rule, the abnormal drop and the cross point in Hall data implies that a possible Lifshitz transition has occurred between 30 K and 60 K, likely driving the compensated electron-hole density, the large MR as well as the metal-semiconductor transition in MoAs_2_. Our results indicate that the family of centrosymmetric transition-metal dipnictides has rich transport behavior which can in general exhibit variable metallic and topological features.

## Introduction

Three-dimensional (3D) topological quantum materials, including topological insulators (TIs)^1^, topological Dirac semimetals (DSMs)^2–6^ and Weyl semimetals (WSMs)^7–14^, have been discovered and intensively investigated recently. These materials exhibit a variety of interesting physical properties, owing to their unique electronic structures and spin textures^2,6,8,10,15^, and thus show a broad application potential. An ideal TI usually exhibits a clear resistivity plateau at low temperatures due to the robust surface states^16–19^. In an ideal WSM, the Weyl fermions disperse linearly all the way across the Weyl nodes which appear in pairs with opposite chiralities by breaking time reversal symmetry (TRS) or inversion symmetry^15,20,21^. The relativistic electronic dispersion and chirality-based topological property result in various semi-metallic transport properties and produce a number of novel phenomena such as the anomalous Hall effect^7^ and the Fermi arcs^9^.

Recently, the WSMs have been theoretically predicted and experimentally discovered in a family of transition-metal pnictides represented by TaAs family^7–15^. Consequently, dozens of topological semimetals showing exotic physical properties were reported and studied in detail, such as ZrSiS(Te)^22,23^, MoTe_2_^24,25^ and WTe_2_^26^, in addition to the TaAs family. All these materials are non-centrosymmetric in the crystal structure and are further classified into two types of Weyl fermions: with or without the Lorentz symmetry in their energy-momentum dispersions. More recently, a new family of topological semimetals (TSMs) which crystallizes in the centro-symmetry monoclinic structure, the transition-metal dipnictides *XPn*_2_ (*X* = Ta, Nb, *Pn* = P, As, Sb) as represented by TaSb_2_, have been discovered^27–29^.

The reported transport properties of the centrosymmetric *XPn*_2_ exhibit the extremely large magnetoresistance (MR) and ultrahigh electronic mobility in common^30–33^. Meanwhile they also exhibit the material’s-dependent low-temperatures resistivity plateau and negative MR^27,30,34^. While the explanations of these features are still complicated, they are likely related to the disentangled bulk electron/hole bands and topological non-trivial surface states as revealed in the electronic band structure calculations^35^. On the other hand, the coexistence of bulk and surface states is appealing for materials applications. We are thus motivated to search for other candidates in this family with potentially more robust surface states and better metallicity.

In this paper, we report a new member of this family, MoAs_2_, which also crystallizes in a monoclinic structure. Our experimental results indicate that MoAs_2_ undergoes a metal-to-semiconductor crossover under the applied magnetic field up to 5 T. In particular, a very clear resistivity plateau is observed below 18 K even in the absence of the magnetic field. The plateau feature is robust against the applied magnetic fields up to 9 T. The MR is relatively large as the applied magnetic field is perpendicular to the current, reaching about 2600% at 2 K and 9 T, but violates the Kohler’s rule below 70 K. When the applied field is rotated from perpendicular to parallel to the current direction, the MR drops rapidly and finally becomes negative. Hall resistivity shows the non-linear field-dependence below 70 K and changes from positive to negative at around 40 K. The two-band model fit yields the abnormal drop in hole-mobility below 30 K and a strange cross point in carrier density at around 60 K. All our experimental results indicate a possible Lifshitz transition occurring between 30 K and 60 K, which is likely a direct factor to drive the compensated electron-hole carrier density, the large MR as well as the metal-semiconductor-like transition in MoAs_2_.

## Results

**Figure 1:**
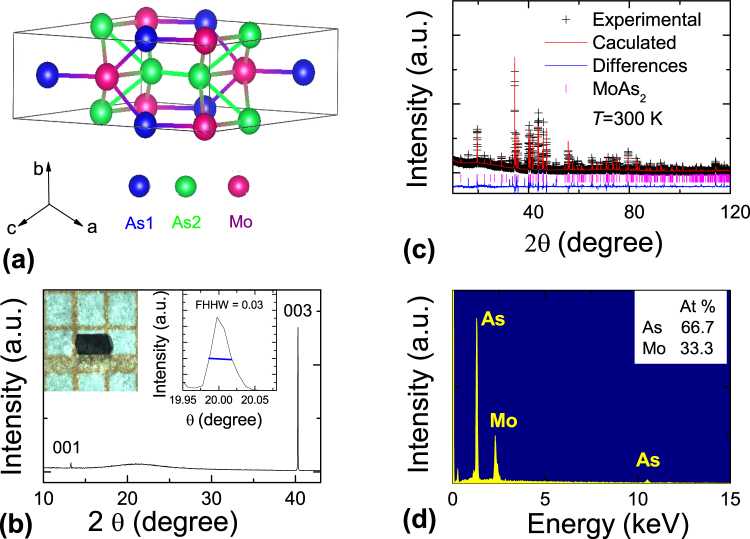
Crystal Structure. (**a**) The crystal structure of MoAs_2_. (**b**) The (00 l) X-ray diffraction patterns of single crystal. Left inset: picture of a typical MoAs_2_ single crystal. Right inset: the rocking curve with a very small half-high-width. (**c**) The Rietveld refinement profile for MoAs_2_ sample at room temperature. (**d**) EDX spectroscopy at room temperature.

Figure 1 shows the detailed information of MoAs_2_ crystal structure. In Fig. 1a, MoAs_2_ crystallizes in a monoclinic structure with C2/m space group, which is common to the *XPn*_2_ family. Figure 1b shows the (00l) peaks of MoAs_2_ single crystal X-ray diffraction, implying that the crystal surface is normal to the c-axis. The b-axis is perpendicular to the ac-plane, and parallel to the current direction. The left inset of Fig. 1b shows a picture of a large polyhedral MoAs_2_ crystal with millimeter dimension. The polyhedral crystal with rectangle-shape is consistent with the monoclinic structure. The right inset shows the X-ray rocking curve with a very small half-high-width, implying the high quality crystal. Figure 1c exhibits the Rietveld refinement profile and its structure parameters in MoAs_2_ at room temperature (more details of crystal structure parameters can be found in Table S1 in Supplementary Information). Through the Rietveld structural analysis, the refined lattice parameters are extracted to be *a* = 9.064(7) Å, *b* = 3.298(7) Å, *c* = 7.718(3) Å, and *β* = 119.37(1)^°^ as reported in the previous literatures^36,37^. Figure 1d shows the EDX data with the Mo and As contents of 33.3% and 66.7%, respectively, consistent with the ratio of Mo and As.

**Figure 2:**
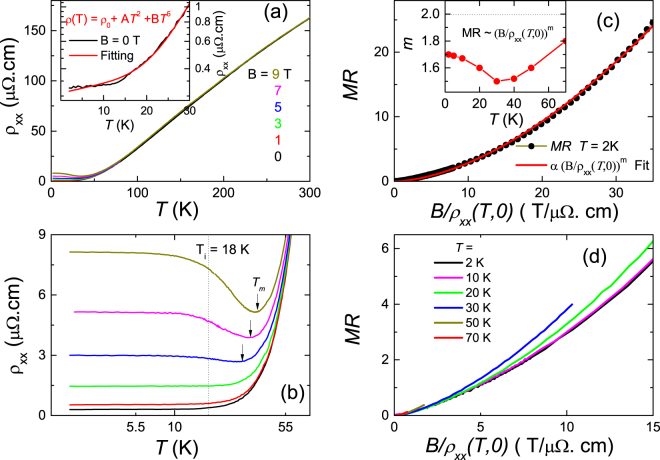
Temperature dependence of resistivity for MoAs_2_ and the Kohler’s rule below 70 K. (**a**) Resistivity of MoAs_2_ as a function of temperature in several magnetic fields (*B* = 1, 3, 5, 7, 9 T.) perpendicular to the current. Inset shows a deviated Fermi liquid behavior below 30 K at B = 0. The (**b**) shows a clear plateau resistivity at low temperature. (**c**) MR vs. B/*ρ*_0_ at 2 K. The red line represents the fitting curve. The inset shows the fitting *m* values at different temperatures. (**d**) The breakdown of Kohler’s rule below 70 K.

Figure 2 displays the evolution of the magneto-resistivity as a function of temperature in MoAs_2_ down to 2 K with the magnetic field ${\bf{B}}\parallel c\perp {\bf{I}}$. As shown in Fig. 2a, the value of *ρ*_*xx*_ at 300 K is about 163 *μ*Ω cm at **B** = 0 T, comparable to that of high quality WTe_2_^26^ and MoTe_2_^24^, the potential candidates of the type-II WSM. While, *ρ*_*xx*_ at 2 K falls to 0.29 *μ*Ω cm, yielding a large value of residual resistivity ratio (RRR) = 562. This confirms the high quality of the crystal. The extraordinarily low residual resistivity at 2 K was previously found in Cd_3_As_2_^38^, ZrSiS^39^ and high purity Bi^40^ with very large RRR.

The zero field resistivity *ρ*_*xx*_ exhibits highly metallic behavior. Upon decreasing temperature, it decreases almost linearly in the high temperature regime. Below 30 K, however, *ρ*_*xx*_ deviates severely from the Fermi liquid behavior. Considering that this contribution to resistivity involves the electron-phonon(e-ph) interaction according to the Bloch-Gruneisen theory^41^. We employed the formula *ρ* = *ρ*_0_ + *aT* ^2^ + *bT* ^5^ to fit the low temperature resistivity as shown in inset of Fig. 2a, where the *ρ*_0_ is the residual resistivity at *T* = 0 K, the terms of *T* ^2^ and *T* ^5^ represent the contributions of the e-e and e-ph scatter, respectively. It is found that resistivity does not exactly match this fit. Instead it remains almost constant below 20 K, displaying a clear resistivity plateau.

An applied magnetic field does hardly change the resistivity in the high temperature regime as compared with that of the zero field. A significant change in *ρ*_*xx*_ appears around *T*_*m*_ = 40 K, where the resistivity starts to saturate for small field or crossovers to the semiconductor behavior for large field. After the crossover the resistivity is soon saturated, developing a robust resistivity plateau in the low temperature regime below *T*_*i*_ = 18 K, defined as the onset temperature of the plateau where ∂^2^ *ρ*(*T*)/∂*T* ^2^ = 0, as clearly observed in the Fig. 2b. The crossover behavior becomes prominent with increasing magnetic field up to 9 T while the crossover temperature does not change too much.

A large MR ( = (*ρ*_*xx*_(*B*) − *ρ*_*xx*_(0))/*ρ*_*xx*_(0)) is observed, reaching 2600% at 2 K and 9 T in Fig. 2c. Although the MR in MoAs_2_ is one or two orders of magnitude smaller than that in other semimetal compounds, it is still far larger than the value in some ferrimagnetic materials^42^. According to the Kohler’s rule^41,43^ (MR ∝ (*B*/*ρ*(*T*, 0))^*m*^), all the MR at various temperatures can be scaled onto a single line with a constant *m* = 1,2. In Fig. 2c, the fit of MR curve at 2 K yields *m* = 1.7. A careful analysis to the MR across the metal-semiconductor-like transition regime found that the *m*, as shown in inset of Fig. 2c, displays a minimum value around 40 K for *m* = 1.5, but then increases slightly to about 1.7 below 10 K. The small *m* value substantially violates the classically field-square dependence of the MR in the semiconductor-like regime. Noted that the exponent *m* (~1.5) has been observed in under-doped cuprates and organic conductor which both are widely considered as non-Fermi liquid system^44,45^. The results, as plotted in Fig. 2d, display that the MR below 70 K obviously deviates from the Kohler’s rule, indicating that the sample is a multi-band system possessing the variable carrier concentrations or the mobility ratio of electron to hole when temperature goes down. Therefore, we suggest that the conventional e-e and e-ph interactions may be hard to explain the appearance of resistivity plateau in MoAs_2_, implying that this intrinsic plateau is closely related to the topological nature as observed in many semimetal materials^27,46^.

However, in these semimetal compounds, saturated resistivity plateaus at much lower temperatures are naturally expected. The microscopic origin of this phenomenon remains debated, depending on the expanded energy scale around which the plateau feature sets in. In the candidates of TIs, SmB_6_^18,19^ and LaSb^46,47^, the resistivity plateau is well-attributed to the topological non-trivial surface states which are robust against disorders. It is interesting that a clear resistivity plateau was also observed in TaSb_2_, a candidate of TSM^27^. Although bulk excitations are involved in this material, the resistivity plateau is plausible due to the surface states which are topological non-trivial in the sense of doped weak TIs^35^. It is remarkable that the resistivity plateau of MoAs_2_ sets in at *T*_*i*_ = 18 K, about four times of that of SmB_6_ (*T* = 5 K)^19^.

**Figure 3:**
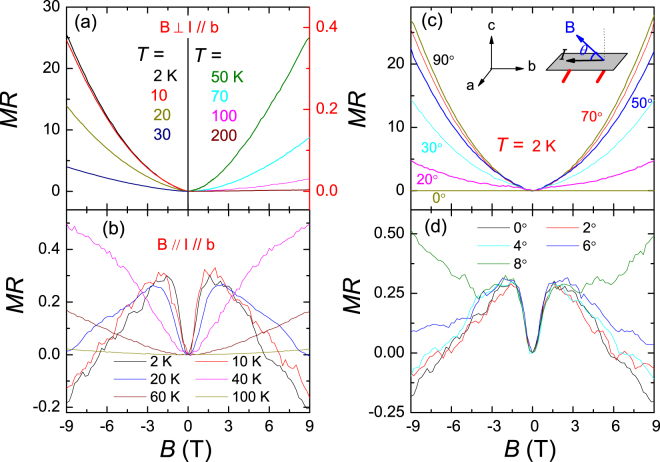
Magnetic field dependence of MR in MoAs_2_ single crystal. (**a**) Magnetoresistance ${\rm{MR}}=({\rho }_{xx}(H)-$ (${\rho }_{xx}\mathrm{(0))}/{\rho }_{xx}\mathrm{(0)}$) versus magnetic fields along the c-axis at different temperatures as ${\bf{B}}\perp {\bf{I}}\parallel {\rm{b}}$. (**b**) MR vs. fields for ${\bf{B}}\parallel {\bf{I}}\parallel {\rm{b}}$. (**c**) MR plotted as a function of magnetic fields at different angles between **B** and **I**. (**d**) The large and unsaturated negative MR emerges in a narrow window of angle around *θ* = 0°.

Now we turn to the MR of MoAs_2_ as shown in Fig. 3. All data here are displayed without a symmetrizing process. In Fig. 3a, a large MR at low temperatures is observed when the field is perpendicular to the current direction. The value of MR does not change significantly until 20 K where it decreases sharply in consistent with the robust resistivity plateau which persists up to 9 T. At fixed temperatures, the MR increases quadratically for low field and almost linear for larger field without saturation, similar to the previously-known semimetallic materials including TaAs(P)^7,14^, NbAs(P)^7,14^ and WTe_2_^26^. Further increasing temperature, its value approaches to 3% at 100 K, and is less than 1% above 200 K at 9 T. This feature is in contrast to the most of semimetals with a relatively large MR even at room temperatures^14,22^.

As the magnetic field is applied along the current direction, ${\bf{B}}\parallel {\bf{I}}$, the MR becomes negative at low temperatures shown in Fig. 3b. These MR curves are overall axial-symmetric around B = 0 T, with only slight noises due to the extraordinarily low resistivity. The absolute value of MR is much smaller than that in the case of ${\bf{B}}\perp {\bf{I}}$. At 2 K, the MR first increases until about 2 T, then decreases monotonously with the field and changes a sign from positive to negative as B ~ 6.5 T, reaching about 20% at 9 T. The negative MR decreases gradually upon heating and disappears above 40 K, beyond which the MR turns back to positive.

Figure 3c shows the field dependence of MR at various angles *θ* of the magnetic field with respect to the current direction at 2 K. By rotating *θ* = 90° → 0°, the MR drops quickly and shows quadratically field-dependence. The negative MR is further illustrated in Fig. 3d when *θ* approaches to 0°. At B = 9 T, the MR is still negative for *θ* ≤ 4°. When *θ* is larger than 4°, the MR recovers positive and increases with *θ*. The negative MR at *θ* = 0° is limited in a narrow window over ~ 6.5 T. The phenomenon of negative MR has been observed in a number of metallic compounds with high mobility of charge carriers. The interpretation of this phenomenon remains debated, too, given the fact that it may appear in both topological trivial and non-trivial materials. A crucial issue behind the negative MR is the current jetting effect due to the field-induced anisotropy. This effect is usually elusive but has been recently suggested as a main cause of negative MR in TaP compound^14,48^. In the TaSb_2_ samples, however, the current jetting effect was shown to play a minor influence on the negative MR by using different contact configurations^27^. In the ideal WSMs, the negative MR could be best-understood due to the chiral anomaly. The similar topological interpretation applies to those with ill-defined Weyl points or Dirac points if they appear in pairs and separate in momentum space^49,50^. In the present compound MoAs_2_, the topological interpretation seems to be consistent with the robust resistivity plateau discussed previously.

**Figure 4:**
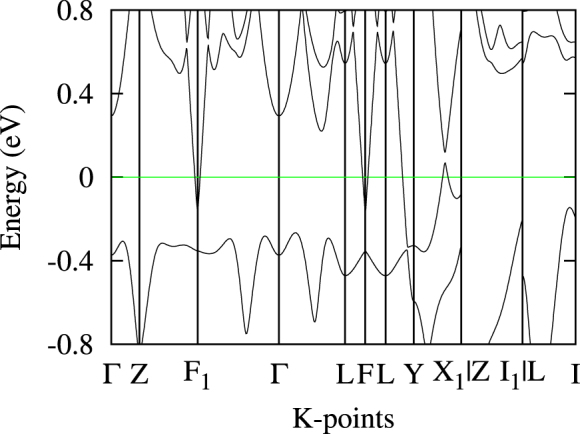
Band structure of MoAs_2_ with SOC.

Figure 4 shows the band structure with inclusion of spin-orbit coupling (SOC). The inversive bands from X_1_ to Y lead to a Dirac cone in the absence of the SOC, while it opens a gap when the SOC is taken into account, resulting in two disentangled and nearly compensated electron/hole bands. We can calculate the topological index for each of these bands according to the parity-check method^51^. Different to TaSb_2_ or other previously-known *XPn*_2_ compounds, the indices of the partially-filled bands of MoAs_2_ are topologically strong (Detailed analysises for the topological nature of the band structure and surface states will be given elsewhere). The result indicates that the surface states in MoAs_2_ are more robust than those in TaSb_2_^27^, consistent with the observed resistivity plateau in our present sample.

**Figure 5:**
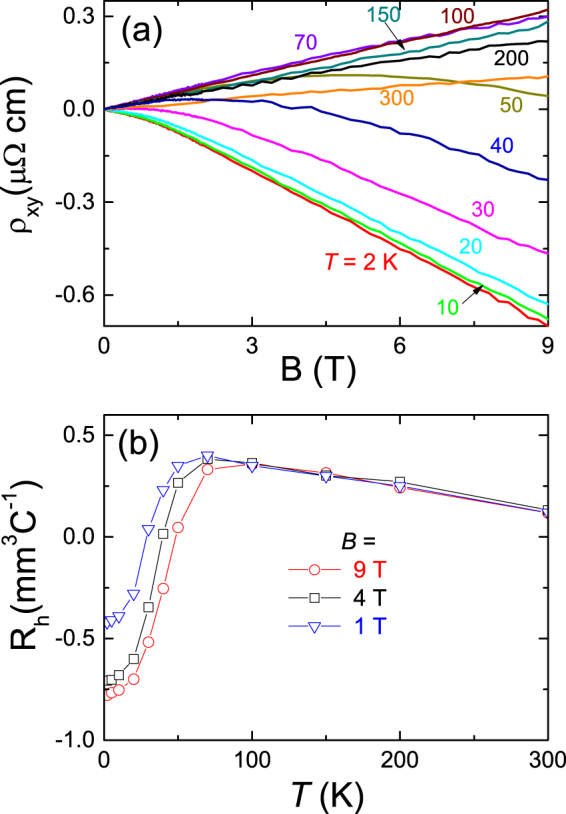
Hall effect for MoAs_2_. (**a**) Magnetic field dependence of Hall resistivity at several different temperatures up to 9 T. (**b**) Hall coefficient vs. temperatures at 1 T, 4 T and 9 T.

**Figure 6:**
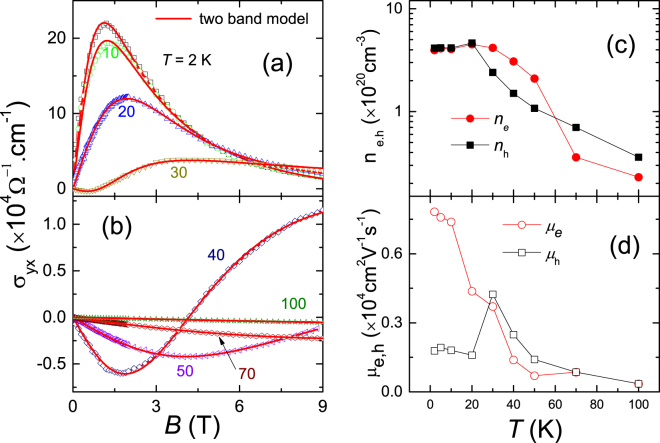
Hall conductivity, the carrier density and mobility. (**a**) and (**b**) Magnetic field dependence of Hall conductivity at several representative temperatures up to 9 T. The red solid lines are the fitting curves using two-band model. (**c**) Electrons and holes densities and (**d**) mobilities as a function of temperature below 100 K.

Figure 5a maps the magnetic field dependence of the Hall resistivity for MoAs_2_ at various temperatures, with the magnetic field ranging from 0 to 9 T. The field dependence of *ρ*_*xy*_(B) is almost linear with positive slope at high temperature. It starts to bend strongly below 70 K. For T ≤ 40 K, *ρ*_*xy*_(B) shows a pronounced sign reversal from positive to negative, and finally recovers the nearly linear behavior, implying a multi-band system in MoAs_2_. Figure 5b shows the temperature dependence of the Hall coefficient at 1 T, 4 T, and 9 T. As temperature decreases, R_*H*_ firstly increases from 300 K, and then undergoes a sharp drop at around 70 K. R_*H*_ changes its sign from positive to negative at around 40 K, implying a partial compensation between the hole-type and electron-type carriers at low temperatures. The sign-change in R_*H*_ is usually (though not always) an important indication of multiple bands, so the feature of the sign-changed R_*H*_ confirms further the multi-band effect in MoAs_2_. The sign-changed R_*H*_ together with the increase of electron-mobility and -density as shown in Fig. 6 implies a possible temperature-induced Lifshitz transition occurring at around temperature, resulting in a drop R_*H*_ below 70 K associated with the decrease of hole pockets.

We carefully analyze the Hall conductivity according to the two-band model^41^. The results of the densities and mobilities of both electrons and holes as a function of the temperature are shown in Fig. 6. In this model, the Hall conductivity tensor is given by:

1 $$\begin{array}{cc}{\sigma }_{xy}=-\frac{{\rho }_{xy}}{{\rho }_{xx}^{2}+{\rho }_{xy}^{2}} & {\sigma }_{xy}=eB[\frac{{n}_{h}{\mu }_{h}^{2}}{1+{({\mu }_{h}B)}^{2}}-\frac{{n}_{e}{\mu }_{e}^{2}}{1+{({\mu }_{e}B)}^{2}}]\end{array}$$

Here, *n*_*e*_(*n*_*h*_) and *μ*_*e*_(*μ*_*h*_) are electrons (holes) carrier densities and mobilities, respectively. The result gives a excellent fit below 100 K and the obtained *n*_*e*,*h*_ and *μ*_*e*,*h*_ as a function of temperature are shown in Fig. 6c and d. Above 70 K, the hole-type carrier dominates, consistent with the positive Hall coefficients as shown in Fig. 5b. With lowering temperature, the electron-type carrier density increases sharply and becomes dominant, resulting in an observed cross point at about 60 K which is very closed to the characteristic temperature (*T*_*m*_) with the sign-changed R_*H*_. As further cooling down temperature, the *n*_*e*,*h*_ increases slowly, and then becomes almost equivalent and saturated. The calculated density of electrons and holes at 2 K is 3.95 × 10^20^ cm^−3^ and 4.13 × 10^20^ cm^−3^, respectively, implying a perfect compensation of semimetal in MoAs_2_. In Fig. 6d, the mobility, *μ*_*e*,*h*_, as a function of temperature increases slightly and displays more or less overlap above 30 K, followed by a huge divergency that *μ*_*h*_ drops suddenly and *μ*_*e*_ increases sharply. Noted that the metal-semiconductor transition occurs at around 40 K, below which the MR starts to increase dramatically. A combination of the drop in mobility, the cross point in *n*_*e*,*h*_ and the sign-changed R_*H*_ points to a possible electronic structure change at around 30–60 K, named a Lifshitz transition, directly driving the compensation of electron-hole carriers density at low temperature and the extremely large MR as well. The similar behavior has been investigated in the discovered WTe_2_ and MoTe_2_ semimetals by the measurement of ARPES^52,53^, Ultrafast transient reflectivity^54^ and Hall effect^55,56^.

Therefore, the possible Lifshitz transition can be suggested in MoAs_2_, which directly derives a compensated electron-hole carriers density, the large MR as well as the metal-semiconductor-like transition. More experiments in MoAs_2_ such as ARPES need be investigated in future to clarify this phase transition, since it is likely to make out the origin of those transport properties and its topological properties.

## Discussion

Presently, the large MR has been discovered and investigated in magnetic multilayers^57^ and semiconductor 2D-electronic systems^58–61^, in which the rearranged magnetic moments and electron-electron interaction likely play a key role. However, the large unsaturated positive MR recently discovered in some nonmagnetic semimetals^4,7,27,38^ seems to be unique and puzzled. The possible mechanisms can be mainly ascribed to the several factors: (1) the linear energy dispersion of Dirac fermions leading to the quantum limit^62^; (2) the compensated electron-hole carrier density^8,26^; (3) the turn on temperature behavior following the Kohler’s rule^43^; (4) other reasons such as protection mechanism^38^. In MoAs_2_, the positive MR shows the almost quadratic field-dependent not linear at low magnetic fields, implying that the quantum limit should not dominate the transport. MoAs_2_ also displays the turn on temperature behavior as B ≥ 5 T, analogous to the other most of semimetals, but its MR clearly violates the Kohler’s rule below 70 K. On the other hand, the sign-changed R_*H*_ and the almost equal amount of electron and hole density at low temperatures have been observed in the present sample. We thus suggest that the large MR can be attributed to the electron-hole compensation, similar to WTe_2_^26^.

Unlike the high mobility of the two-dimensional electron gas (GaAs/AlGaAs heterostructure)^63^, the negative MR associated with the chiral anomaly has been viewed as a key transport signature in semimetal compounds, such as TaSb_2_^27^, TaAs_2_^30^, and Cd_2_ As_3_^64^. When the magnetic field is parallel to the electric current, the chiral anomaly induces the non-conservation of the fermions number with a given chirality, resulting in the negative MR. Recently, an alternative scenario for the negative MR has been suggested, called as the current jetting effect which is associated with an inhomogeneous current distribution inside the sample in a magnetic field^14,48^. We thus perform the different contact configurations on resistivity measurements (see the Fig. S1 in Supplementary Information). Consequently, the negative MR is robust and shows weakly dependent on the different contact configurations. Therefore, we believed that the current jetting effect may play a minor role in the negative MR in our present sample, as far as in the XPn_2_ system^27,30^.

In summary, we reported an iso-structural MoAs_2_ compound which crystallizes in the monoclinic structure with a centrosymmetric space group C2/m. It shows large positive MR and undergoes a metal-semiconductor crossover under 9 T, but clearly violates the Kohler’s rule below 70 K. At low temperatures, a robust resistivity plateau with T_*i*_ = 18 K is observed up to 9 T, which is likely caused by the topological non-trivial surface states whose existence should be further examined by the ARPES experiment. When ${\rm{B}}\parallel {\rm{I}}$, the negative MR is observed regardless of the different contact configurations, implying a minor current jetting effect in our sample. On the other hand, The analysis of two-band model in Hall resistivity indicates an almost equal amount of electrons and holes at low temperature, consistent with the linear Hall resistivity. A combination of the drop in hole-mobility, the cross point in density and the sign-changed *R*_*H*_ as a function of temperature together with the breakdown of Kohler’s rule suggests a possible Lifshitz transition occurring between 30 K and 60 K, likely driving the electron-hole compensation, the large MR as well as the metal-semiconductor-like transition in MoAs_2_.

## Methods

Very high quality single crystals of monoclinic MoAs_2_ were grown through chemical vapor transport reaction using iodine as transport agent. Polycrystalline samples of MoAs_2_ were first synthesized by solid state reaction using high purified Tantalum powders and Antimony powders in a sealed quartz tube at 973 K for three days. Subsequently, the final powders together with a transport agent iodine concentration of 10 mg/cm^3^ were ground thoroughly, and then were sealed in a quartz tube with a low Vacuum-Pressure of ≤ 10^−3^ Pa. The single crystals MoAs_2_ were grown in a horizontal tube furnace with a temperature gradient of 120 °C between 1120 °C − 1000 °C for 1–2 weeks. The high quality single crystals with apparent monoclinic shape were picked from the resultant.

X-ray diffraction patterns were obtained using a D/Max-rA diffractometer with CuK α radiation and a graphite monochromator at the room temperature. The single crystal X-ray diffraction determines the crystal grown orientation. The composition of the crystals was obtained by energy dispersive X-ray (EDX) spectroscopy. No iodine impurity can be detected in these single crystals. The stoichiometric ratio is fairly homogenous. The (magneto)resistivity and Hall coefficient measurements were performed using the standard four-terminal method in which the current is parallel to the b-axis. Ohmic contacts were carefully prepared on the crystal with a Hall-bar geometry. Silver wires were attached on the surface of crystals with silver paste. The low contact resistance was obtained after annealing at 573 K for an hour. The physical properties were performed in a commercial Quantum Design PPMS-9 system with a torque insert with a temperatures range from 2 to 300 K and the magnetic fields up to 9 T. The density functional theory calculations of MoAs_2_ were performed by employing the plane wave basis projector augmented wave (PAW) method implemented in the Vienna ab-initio Simulation Package^65,66^. The electronic structure calculations were then performed using crystal structures with optimized lattice constants and internal atomic parameters.

## Electronic supplementary material

Supplementary Information
