# Supplementary material for: Magnetoresistance and robust resistivity plateau in MoAs2
Source: Sci Rep. 2017 Nov 15;7:15669. doi: 10.1038/s41598-017-15962-w (PMC5688174; doi:10.1038/s41598-017-15962-w)
Supplement: Supplementary file 1 — Supplementary Information [file 41598_2017_15962_MOESM1_ESM.pdf]

SUPPLEMENTARY INFORMATION

Magnetoresistance and robust resistivity plateau in $MoAs_2$

Jialu Wang[1], Lin Li[1], Wei You[1], Tingting Wang[1], Chao Cao[1], Jianhui Dai[*,1†], Yuke Li[*,1*]

[1] *Department of Physics and Hangzhou Key Laboratory of Quantum Matters, Hangzhou Normal University, Hangzhou 310036, China*

† daijh@hznu.edu.cn

* yklee@hznu.edu.cn

TABLE S-I. Crystallographic data of $MoAs_2$ at room temperature are derived from Rietveld refinement based on space group C2/m. a = 9.064(7) Å, b = 3.298(7) Å, c = 7.718(3) Å, and $\alpha = \gamma$ = 90° and β = 119.37(1)°; R_{wp} = 14.03, χ^2 = 1.84

.

Atom	x	y	z
Mo	0.1533(7)	0	0.2006(1)
As1	0.1464(4)	0	0.5334(5)
As2	0.4063(1)	0	0.1085(7)

I. CRYSTAL STRUCTURAL PARAMETERS AND NEGATIVE MAGNETORESISTIVITY

Fig.S1 shows the different contact configurations of magnetoresistivity measurements in $MoAs_2$. The negative MR is clearly observed when the magnetic field is parallel to the current regardless of the contact configurations. The MR decreases gradually but remains negative until θ over 6° for two kinds of contact configurations. The difference in the measured MR between contact 1 and contact 2 is about 10%, which is much smaller than those of TaP sample as reported in previous literature[1]. Therefore, the negative MR in $MoAs_2$ is roust and the current jetting effect should be a minor effect for the appearance of the negative MR in our present sample.

[1] F. Arnold, et al., Nat. Commun. **7** 11615 (2016).

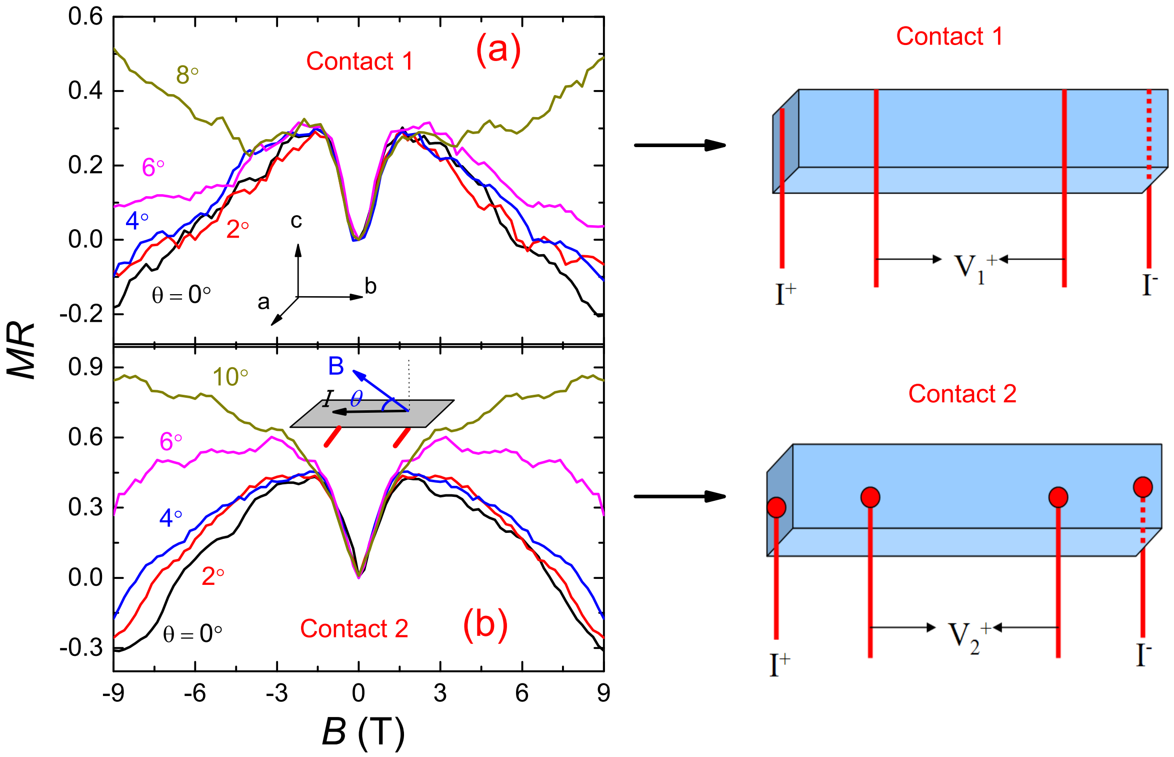

Figure S1. **The negative MR of $MoAs_2$ are observed with two kinds of resistivity contact configurations.** Left: (a) The clear negative MR at several different angles is observed. (b) as a comparison, the negative MR with another contact configuration is shown. Right: the sketch map of resistivity measurement for contact 1 and contact 2, respectively.
